# Ethnic differences in parathyroid hormone secretion and mineral metabolism in response to oral phosphate administration

**DOI:** 10.1016/j.bone.2009.04.237

**Published:** 2009-08

**Authors:** Liya Yan, Inez Schoenmakers, Bo Zhou, Landing M. Jarjou, Emily Smith, Shailja Nigdikar, Gail R. Goldberg, Ann Prentice

**Affiliations:** a MRC Human Nutrition Research, Elsie Widdowson Laboratory, Fulbourn Road, Cambridge CB1 9NL, UK; b Department of Preventive Medicine, Shenyang Medical College, 146 Huanghe North Street, Shenyang 110034, PR China; c MRC Keneba, Gambia

**Keywords:** Phosphate, Parathyroid hormone, Bone markers, Bone and mineral metabolism, Ethnic differences

## Abstract

Ethnic differences in bone metabolism have been reported and it has been suggested that these may be partly due to prolonged exposure to an elevated plasma parathyroid hormone (PTH) concentration or a decreased sensitivity to PTH. We explored ethnic differences in bone and mineral metabolism by 5 days of oral phosphate (P) loading to stimulate PTH secretion. Healthy older people from UK (B), The Gambia (G) and China (C), 15 individuals from each sex and ethnic group, were studied. Blood and urine samples were collected before and 2 h after P dose on days 1, 4 and 5 and on a control day. The induced changes (%) in PTH and markers of mineral and bone metabolism after 2 h and over 5 days were examined.

At baseline, PTH, 1,25(OH)_2_D and bone turnover markers were higher in Gambian subjects than in British and Chinese subjects (*P* ≤ 0.01).

2 h after P loading, ionized calcium (*i*Ca) decreased and PTH and plasma P (P) increased in all groups (*P* ≤ 0.01, n.s. between groups). Urinary P to creatinine ratio (*u*P/Cr) increased, the increase being greater in Chinese subjects than in British and Gambian subjects on days 4 and 5 (*P* ≤ 0.01). By day 5, fasting *i*Ca was decreased and P increased in British and Gambian (*P* ≤ 0.01) but not in Chinese subjects. Fasting PTH and *u*P/Cr increased in all groups. There were ethnic differences in changes in bone markers, but the relationship with changes in PTH was comparable between groups.

In conclusion, ethnic differences in mineral metabolism in response to 5-day P loading were found. Chinese subjects showed a more rapid renal clearance of P than British and Gambian counterparts and there were differences between the groups in the skeletal response to P loading, but no evidence was found for resistance to the resorbing effects of PTH.

## Introduction

Populations differ in fragility fracture risk. Differences in the extent of loss of bone quantity and material properties during ageing result from complex interactions between hormone status, genetic traits, diet and physical activity over the lifespan. Increased parathyroid hormone (PTH) secretion and bone turnover induced by poor vitamin D status and/or low calcium (Ca) intake is considered to increase the risk of osteoporosis in older people [1].

However, we have shown that older Gambian and Chinese populations with a low incidence of osteoporotic fractures have elevated plasma PTH associated with low Ca intake and/or poor vitamin D status [2–5]. In the UK Ca intake is close to recommendations, there is a winter nadir in vitamin D status but no corresponding increase in PTH concentration [5,6]. In The Gambia, vitamin D status is good because there is ample opportunity for cutaneous vitamin D synthesis throughout the year, but Ca intake is exceptionally low (∼ 300 mg/day) and PTH concentrations are elevated compared to British adults throughout the year [3,7]. In northern China, vitamin D status is poor in winter, Ca intake is low, and PTH concentrations are higher than those of British counterparts [5]. Furthermore, in older Chinese adults, there is no significant inverse association between PTH concentration and bone mineral status (BMC and BMD), in contrast to Caucasian adults in Britain and elsewhere [5,8]. Resistance to the bone resorbing effects of PTH and racial differences in PTH dynamics have been reported in Black American women [9–11]. All these findings have led us to hypothesise that prolonged exposure to elevated PTH in plasma, either chronically or seasonally, may alter skeletal responsiveness to its effects [12].

Oral phosphate (P) has been used by others to stimulate PTH secretion and investigate the effect of PTH on bone turnover [13], to test if skeletal responsiveness to PTH differs between ethnic groups. We conducted a study in healthy older adults in the UK, The Gambia and China using a similar protocol.

## Subjects and methods

### Subjects

The study was conducted at MRC Human Nutrition Research (HNR), Cambridge, UK, MRC Keneba, The Gambia, and Shenyang Medical College, Shenyang, PR China. Cambridge (latitude 52°N) is a university town in the southeast of England with a temperate climate. Keneba (latitude 13°N) is a rural village in The Gambia and the climate is tropical, hot and sunny all year with a wet season from June to November. Shenyang (latitude 42°N) is an industrial city in the northeast of China where winter (November–March) is cold and dark but summer (June–September) is hot and sunny.

Thirty healthy adults (15 men, 15 women) aged 60–75 years were recruited in each centre. Exclusion criteria were any pathological disorder or medication known to affect mineral or bone metabolism. All subjects were ambulatory with normal renal function (fasting plasma creatinine < 115 μmol/l). Subjects in Cambridge (all were of Caucasian origin) with a hip bone mineral density (BMD) < − 2.5 T-score were excluded. This criterion was not used in Keneba and Shenyang because there are no appropriate Chinese and Gambian reference data.

BMD was measured using dual-energy X-ray absorptiometry (DXA) (GE Lunar, WI, USA, software 4.7e in all centres). Height and weight were measured, from which the body mass index (BMI) was calculated. To allow for possible effects of season, subjects were studied once in February–April and once in July–September. Five British (2 males, 3 females), 2 Chinese (1 male, 1 female) and 1 Gambian (female) subjects were not available for the second phase.

Ethical approval was given by the Cambridge Local Research Ethics Committee, the Gambian Government/MRC Laboratories Joint Ethics Committee and the Academic Committee of Shenyang Medical College. Informed written consent was obtained from all subjects.

### PTH stimulation test

**Fig. 1:**
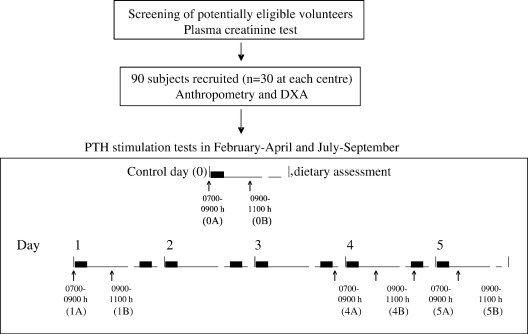
Study design. (■) Water on control day or 1 g elemental phosphate dose during the 5-day test. (↑) Blood and urine samples obtained before (A) and 2 h after (B) water or the morning phosphate dose.

In each phase of the study, subjects took a 1 g dose of elemental P dissolved in 200 ml water, twice daily (0700–0900 h and 1900–2100 h) for 5 consecutive days (Phosphate-Sandoz, HK Pharma, UK) (Fig. 1). Each P dose also contained 246 mg potassium and 938 mg sodium. The stimulation test was preceded within 5 days by a control day in which 200 ml water only was given (day 0). On days 0, 1, 4, and 5, subjects attended the research centre between 0700–0900 h after an overnight fast. Individual subjects attended at the same time for each of their visits. Blood and urine samples were collected on arrival (timepoint A) and 2 hours (2 h) after consuming the water or morning P dose (timepoint B). Subjects remained sedentary during the 2 h and drank 500 ml water to standardise fluid intake but no other food or liquid was consumed.

Dietary Ca and P intakes were assessed in each phase of the study [7,14,15]. Subjects were requested to keep to their usual diet the week before and during each stimulation test.

### Sample collections and laboratory analyses

Blood samples were collected into tubes containing EDTA or lithium-heparin. Blood ionized Ca (*i*Ca) was measured in the lithium-heparin sample (ABL77, Radiometer Medical, USA) within 10 min and pH 7.4 corrected values were used. The remainder of the sample was placed on ice, the plasma was separated from cells within 30 min in a refrigerated centrifuge, and stored at − 80 °C. Urine samples were collected immediately after blood collection. Acidified (HCl, 10 ml/l, laboratory reagent grade, SG 1.18, Fisher Scientific) and non-acidified aliquots were stored at − 20 °C. All samples from The Gambia and samples for analysis of vitamin D metabolites from China were transported on dry ice to Cambridge.

EDTA-plasma was used for analyses of PTH, total N-terminal propeptide of type 1 procollagen (P1NP), N-mid osteocalcin (OC) and β-form of cross-linked C-telopeptide of type 1 collagen (CTX_β_). Lithium-heparin plasma was used for all other analyses. Singleton measurements were made for blood *i*Ca and for those markers analyzed by Elecsys (see below). All other analyses were conducted in duplicate. For each analyte, all samples from a single subject were analyzed in the same run. Plasma total Ca, P and creatinine (Ca, P and Cr), and urinary Ca, P and Cr (*u*Ca, *u*P and *u*Cr) was measured by colorimetric methods (Cambridge: Konelab, Finland; Shenyang: 7170A Hitachi automatic analyzer, Japan). Within and between-assay coefficients of variation (CV) were < 2.0% and < 4.0% respectively. PTH, P1NP, OC and CTX_β_ were measured on an automatic analyzer (Elecsys 2010, Roche Diagnostics, USA). Between-assay CV were 4.7%, 3.2%, 3.1% and 4.3% respectively. The OC assay detects both the intact molecule and N-terminal fragment. Plasma bone alkaline phosphatase (BALP) and urinary deoxypyridinoline (*u*DPD) was measured by ELISA (Metra BAP and DPD EIA kits, Quidel Corporation, USA). Within and between-assay CV were 2.1% and 7.9% for BALP and 6.3% and 10.0% for DPD. Plasma 25- (25-OHD) and 1,25-dihydroxyvitamin D (1,25(OH)_2_D) were measured by radioimmunoassay (DiaSorin, Stillwater, MN, USA and IDS, Tyne and Wear, UK respectively). Within and between-assay CV were 4.1% and 6.1% for 25-OHD, and 7.5% and 9.0% for 1,25(OH)_2_D. Concentrations of *u*Ca, *u*P and *u*DPD were expressed as a ratio relative to urinary creatinine (*u*Cr) to adjust for urine volume (*u*Ca/Cr, *u*P/Cr and *u*DPD/Cr).

Assay performance was monitored using kit and in-house controls. The quality of 25-OHD, 1,25(OH)_2_D and PTH assays was monitored by participation in the Vitamin D External Quality Assessment Scheme (www.deqas.org), and the National External Quality Assessment Scheme (www.ukneqas.org.uk). Cross-calibrations were conducted for those assays that were performed in different centres by measuring in-house control materials, ten aliquots for each analyte. The difference between the laboratories ranged from 1% to 9%. Gambian and Chinese values were adjusted to Cambridge values using the appropriate multiplication factor.

### Data-handling and analysis

Statistical analysis was performed using the Linear Model facility in Data Desk 6.1.1 (Data Description, Ithaca, NY, USA). To permit the exploration of proportion relationships, variables were converted to natural logarithms whereby group differences × 100 correspond closely to percentage differences [(difference / mean) × 100] [16]. All percent differences (± SE) presented were obtained this way. Baseline values are presented as mean (± SD) for normally-distributed data, and as geometric means (95% CI) for skewed data.

The absolute and relative (percentage) changes in each analyte were examined to investigate the response to P administration. Because the patterns of change were similar, only percentage (%) changes are presented for simplicity. The change by 2 h was defined as the change in a variable from timepoint A (before P dose) to timepoint B (2 h after) on the same day. The response on day 0 was used to control for any change that was independent of the effect of administered P. Thus, change by 2 h (%) = (lnB_day (1, 4 or 5)_ − lnA_day (1,4 or 5)_) − (lnB_day 0_ − lnA_day 0_). Change by day 5 (%) was defined as the difference in fasting values between baseline (mean of timepoint A on days 0 and 1) and day 5 (timepoint A), calculated as lnA_5_ − lnA_baseline_. The estimated glomerular filtration rate (eGFR) was calculated according to the Cockroft and Gault equation (Traynor et al., 2006) [39] and the ratio of the maximum tubular reabsorption rate of phosphate to glomerular filtration rate (TmP/GFR) was calculated using the formula described by Payne [18] and Barth et al. [17].

Within groups, the significance of change from baseline by 2 h and day 5, and the difference in 2 h change between days 1 and 5 was examined by Student's paired *t*-test. Differences between groups were examined using ANOVA or ANCOVA. Subject, sex, season and age were independent variables and subject was nested by sex and group. Scheffé post-hoc tests were used to test significance of differences between groups in pairs and to minimise the effects of multiple testing. Multiple linear regression was used to examine the relationship between change in PTH and change in markers of mineral and bone metabolism. Group differences in Ca concentration were examined after adjusting for albumin concentration by including it as an independent variable. All statistical models were set up in a standardised way by generating a full model, which included all potential confounders. Any variable of *P* > 0.05 was removed by backward elimination to provide a parsimonious model. *P* values ≤ 0.05 were considered significant.

## Results

No significant seasonal effects were detected in the British or Gambian groups. A significant difference between the two seasons was found in some of the biochemical markers in the Chinese group. However, this did not affect the interpretation of the data presented in this paper and is not described further. There were significant differences between men and women in many baseline characteristics. However, there were no significant group–season or group–sex interactions identified for intra-individual changes by 2 h and day 5 in any biochemical marker. Therefore, except for subject characteristics and summary information at baseline, the data for each group are presented with season and gender combined.

### Subject characteristics and baseline data

Age, height, weight, BMI, dietary Ca and P intake and hip BMD are indicated in Table 1. There were significant differences in dietary intakes of Ca and P within sex and between groups. Dietary Ca and P intakes and Ca:P ratio were higher in British than Chinese and Gambian subjects (ANOVA *P* ≤ 0.01). Ca and P intakes were lower in Gambian than Chinese subjects (*P* ≤ 0.05). BMI was higher in British than in Gambian subjects (ANOVA *P* ≤ 0.001) and higher in Chinese males than Gambian males (ANOVA *P* ≤ 0.05).

Compliance as estimated by pill count was 100%. No side effects were reported, except for mild to moderate diarrhoea in 3 subjects.

There were significant differences in baseline P, Cr, TmP/GFR and *u*Ca/Cr and in the concentrations of other biochemical markers, most notably, 25-OHD, 1,25(OH)2D and PTH (Table 2). 25-OHD was higher in British than Chinese females, and in Gambian than Chinese males and females. 1,25(OH)2D, PTH and bone turnover markers were higher in Gambian men and women than both British and Chinese men and women (ANOVA *P* ≤ 0.01).

### Change by 2 h

**Fig. 2:**
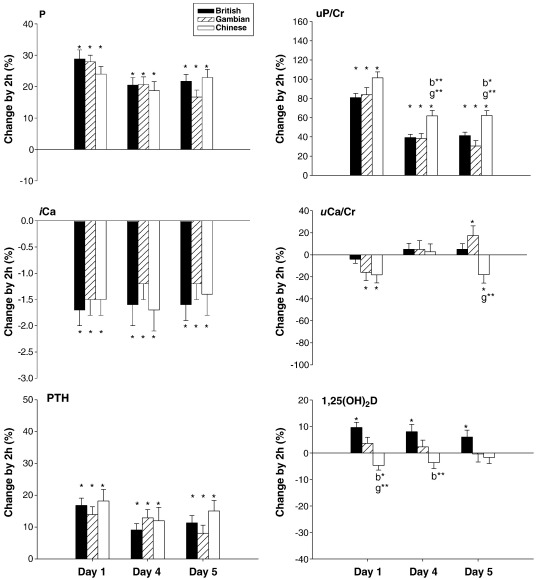
Change by 2 h (lnB_day (1, 4 or 5)_ − lnA_day (1,4 or 5)_) − (lnB_day 0_ − lnA_day 0_)] on days 1, 4 and 5 [% (SE)]. Black, striped and clear bars indicate British, Gambian and Chinese subjects respectively. ⁎ = significant change compared to baseline within country *P* ≤ 0.05. b⁎ and b⁎⁎ = vs. British subjects *P* ≤ 0.05 and *P* ≤ 0.01 respectively; g⁎⁎ = vs. Gambian subjects *P* ≤ 0.01 by Scheffé post-hoc tests in ANOVA.

On day 1, oral P loading resulted in significant increases in P, PTH and *u*P/Cr of comparable magnitude, and concurrent decreases in *i*Ca and *u*Ca/Cr in all three groups, except for *u*Ca/Cr in the British group which was not significantly altered (Fig. 2). On days 4 and 5, the pattern of response was similar to that on day 1, although the magnitude of some changes was smaller (Fig. 2). The exception was *u*Ca/Cr which was increased on day 5 in the Gambian group but decreased in the Chinese group. Significant between-group differences were found in the 2 h change in *u*P/Cr and *u*Ca/Cr (Fig. 2). The increase in *u*P/Cr was higher in the Chinese than in British and Gambian subjects on days 4 and 5 (ANOVA *P* ≤ 0.001) with a trend in that direction on day 1 (ANOVA *P* = 0.1).

The 2 h change in 1,25(OH)_2_D differed between the groups (Fig. 2). In British subjects, 1,25(OH)_2_D was significantly and consistently increased after the P dose on all days. However, it was slightly increased or remained unchanged in the Gambian, whereas in the Chinese subjects, there was a trend for a decrease on all days (ANOVA *P* ≤ 0.001).

Significant changes by 2 h in bone remodelling markers were observed. OC and CTX_β_ increased in the British (2–4% and 8–12% respectively, *P* ≤ 0.05 on all days) and Gambian (2–5% and 2–8% respectively, *P* ≤ 0.05 on day 1) groups and non-significant increases in the Chinese group (3–5% and 6–10% respectively). There were no significant between-group differences. No significant changes were found in any other bone remodelling markers (P1NP, BALP, *u*DPD/Cr, not shown).

### Change by day 5

**Fig. 3:**
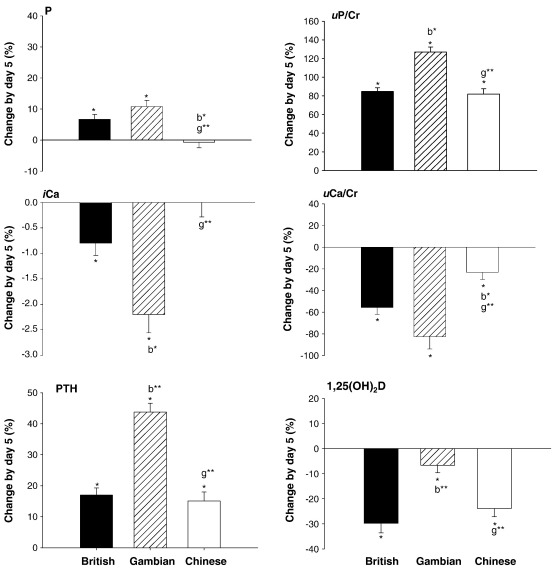
5-day change (lnA_5_ − lnA_baseline_) in fasting samples [% (SE)]. ⁎ = significant change compared to baseline within country *P* < 0.05. b⁎ and b⁎⁎ = vs. British subjects *P* ≤ 0.05 and *P* ≤ 0.01; g⁎⁎ = vs. Gambian subjects *P* ≤ 0.01 by Scheffé post-hoc tests in ANOVA.

There was a significant increase in fasting values of P, PTH and *u*P/Cr and a concurrent decrease in *i*Ca and *u*Ca/Cr in the British and Gambian subjects on day 5 in response to P loading (Fig. 3). In the Chinese subjects, the changes in PTH, *u*P/Cr and *u*Ca/Cr paralleled those in the other two groups, whereas P and *i*Ca on day 5 were not significantly different from baseline (significant between-group difference ANOVA *P* ≤ 0.001). The changes in *i*Ca and PTH were significantly greater in the Gambian group (ANOVA *P* ≤ 0.001). The decrease in *u*Ca/Cr was smaller in the Chinese group (ANOVA *P* ≤ 0.001). Fasting 1,25(OH)_2_D decreased by day 5 in all three groups, the decrease was smaller in Gambian than in British and Chinese subjects (ANOVA *P* ≤ 0.01).

**Fig. 4:**
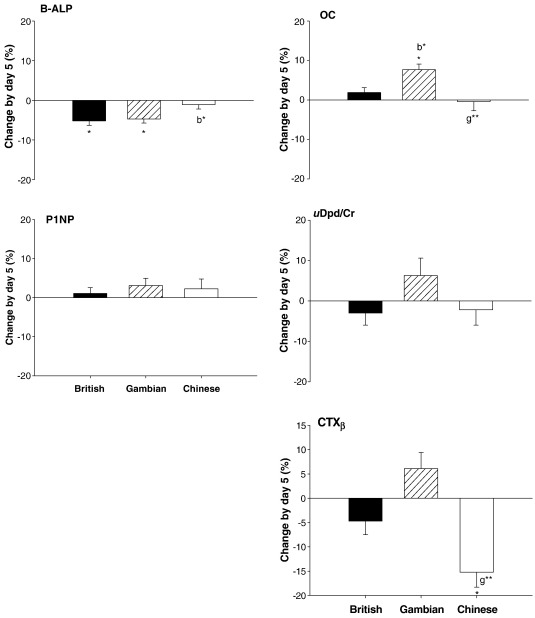
5-day change (lnA_5_ − lnA_baseline_) in bone turnover markers [% (SE)]. ⁎ = significant change compared to baseline within country *P* ≤ 0.05. b⁎ = vs. British subjects *P* ≤ 0.05; g⁎⁎ = vs. Gambian subjects *P* ≤ 0.01 by Scheffé post-hoc tests in ANOVA.

Fasting values of BALP decreased significantly by day 5 in the British and Gambian but not the Chinese subjects (ANOVA *P* = 0.02). P1NP did not change significantly in any group. The change in OC was greater in the Gambian than both the British and Chinese subjects (ANOVA *P* ≤ 0.01). There were no significant changes in any of the markers of bone resorption, except for CTX_β_, which decreased in the Chinese subjects (Fig. 4, ANOVA *P* ≤ 0.001).

Regression analyses revealed that the 5-day change in PTH was significantly and positively related to the changes in CTX_β_ and OC with no significant group interaction (*P* ≤ 0.001 and *P* = 0.002 respectively). No significant association with PTH was found for any of the other markers.

## Discussion

As expected, oral P stimulated PTH secretion in all British, Gambian and Chinese subjects. However, the induced perturbations in Ca and P homeostasis produced different responses by the kidney and skeleton. In particular, the increase in PTH was more pronounced in the Gambian group, and the Chinese subjects had more rapid renal P clearance and a greater renal Ca conservation. In addition, the Chinese subjects showed very little change in bone turnover markers, as evidenced by fasting values on day 5, in contrast to the changes in bone formation and/or resorption markers observed in the other groups.

We assume that the differences in P metabolism were not due to differences in intestinal absorption efficiency because dietary P is usually well absorbed (55–80%) and plasma P increases within hours after ingestion [19]. Indeed a significant increase in P was observed 2 h after the P dose in all groups. Plasma P is predominantly regulated by changes in urinary P excretion [20] and consistent with this, *u*P/Cr was increased in all three groups. However, in the Chinese subjects the increase by 2 h was significantly greater than in the British and Gambian group. This higher *u*P/Cr may explain why fasting P on day 5 was not increased in the Chinese group compared to baseline, in contrast to the increase observed in British and Gambian subjects. We speculate that the more rapid P clearance in Chinese subjects may be due to differences in renal regulation of P metabolism through a mechanism that is independent of PTH, for example fibroblast growth factor 23 (FGF23) or gastro-intestinal factors[21,22]. Plasma FGF 23 is reported not to respond to changes in P absorption within a few hours [23]. The observed changes 2 h after P administration might rather arise from modulation of the renal sodium-phosphate co-transporter by the plasma P concentration and/or through at present unrecognized signals involved in the phosphate intestinal-renal axis and emanating from the gastro-intestinal tract in response to an increase in P ingestion or absorption [22,24].

Our results may reflect an adaptation of the kidney and/or skeleton to a low Ca but sufficient P intake in older Chinese adults, and suggests a mechanistic explanation for our previous findings that a higher endogenous PTH is not associated with lower bone mineral status or higher bone resorption [5]. The data suggest that the roles of the kidney and skeleton in the regulation of Ca and P metabolism may have shifted towards a greater role for the kidney, more efficiently clearing P and reducing urinary Ca losses. It is impossible to tell from these data whether this was caused by skeletal resistance to the action of PTH or increased renal sensitivity to PTH, or to P loading. Furthermore, the combined changes in PTH (increase) and 1,25(OH)_2_D (decrease) may have resulted in preventing bone resorption or reducing bone formation [25] in the Chinese group. A different pattern was seen in the Gambian group. The extremely low dietary Ca intake (Table 1) is close to factorial calculations of obligatory Ca losses in the literature [26] and may have prevented the ‘adaptation’ seen in the Chinese group, because maintenance of plasma Ca concentration needs fractional Ca absorption and renal Ca conservation to be at their physiological maximum, which requires 1,25(OH)_2_D to be high all the time. These different responses may explain the differences in bone turnover markers. These remained unchanged in the Chinese group, with the exception of CTX_β_, which showed a significant decrease by day 5.

The 5-day increase in fasting PTH also differed between groups. The greater decrease in *i*Ca, and correspondingly greater increase in PTH in the Gambian subjects may be due to their habitual exceptionally low Ca intake and the low Ca:P ratio in the intestine after P loading. This would have led to relatively less Ca and more P being absorbed. The significant decrease in BALP by day 5 in the British and Gambian subjects may be due to inhibition of bone formation by PTH and/or a reduction in 1,25(OH)_2_D, as reported in other P and 1,25(OH)_2_D loading studies [27,28]. This contrasts with the current concept of endogenous or exogenous PTH stimulating bone formation [29] and the observed tendency of an increase in P1NP. Potentially, this might be attributed to the different phases of osteoblast differentiation and activation, reflected by these markers [30]. The significant increase in OC (a marker of bone formation and potentially resorption [31]) and a tendency towards an increase in CTX_β_ and *u*DPD/Cr in the Gambian subjects may be attributed to their more pronounced increase in PTH.

Despite the decrease in CTX_β_ in the Chinese group multiple linear regression analysis demonstrated that the changes in CTX_β_ and OC were positively related to the change in PTH and that the relationships did not differ significantly between the groups. These findings therefore provide no evidence in support of differences in skeletal resistance to PTH between the groups. Our results in The Gambia are therefore different to those in African-Americans in which skeletal resistance to PTH was found; a smaller increase in bone resorption compared with White counterparts in response to PTH infusion [9,10]. The differences between the studies may be due to subjects' genotype, body composition, diet, life-style and/or different methods used to stimulate PTH secretion (PTH or citrate/Ca infusion over 24 h vs. oral P over 5 days) [9,10]. In particular, the habitual low calcium intakes of Gambian subjects, leading to secondary hyperparathyroidism [3,7] and potential enlargement of the PTH reserve [32], the relatively high Vitamin D status throughout the year and the high levels of physical activity and low BMI in Gambians compared to African-Americans [9,10] may have influenced these results. This was reflected in baseline characteristics; Gambian subjects had elevated PTH, 1,25(OH)_2_D and bone turnover markers compared to the British and Chinese subjects. We have previously reported elevated PTH, 1,25(OH)_2_D and markers of bone formation, but not resorption, in a study of Gambian women [3], in which DPD output measured in 24 h urine samples was lower compared to British counterparts. In the present study early morning fasting urine samples were collected and DPD was expressed as *u*DPD/Cr. This might, to some extent, explain the discrepancy between our studies.

The 2 h change in 1,25(OH)_2_D in response to P loading was a decrease in the Chinese, and an increase in the British and Gambian subjects. This may have been due to differences in plasma P and renal P filtration rate which suppresses 1,25(OH)_2_D production [33]. In contrast, the 5-day change in 1,25(OH)_2_D was a decrease in all groups. The magnitude of the decrease was significantly smaller in the Gambian compared to the British and Chinese subjects. This may be due to the greater 5-day increase in PTH in the Gambian subjects which would have had a greater stimulatory effect on renal 1-α hydroxylase activity [12], and therefore counteracted the effect of the increased absorbed P load.

There are some limitations to this study. Firstly, it was conducted in subjects consuming their habitual diet. The mean Ca and P intakes in the three groups were very different, which may have confounded the changes in PTH and mineral metabolism in response to the same absolute amount of P given. However, standardisation of diets across groups would have led to deviations from habitual intake and induced changes in ‘usual’ calcium and bone metabolism. Secondly, the range of changes in PTH and bone markers induced may have been too small or too short to fully evaluate skeletal responsiveness to PTH. However, the changes in plasma P and PTH were close to circadian variations [34,35] and therefore probably correspond with variations in plasma P, *i*Ca and PTH induced by diet, physical activity or circadian cycle. The urinary excretion of Ca, P and DPD were expressed as a ratio relative to *u*Cr to adjust for urine volume. Therefore, they may depend on kidney function, lean body mass (LBM) or BMI and potentially on diet, fluid intake and sweat losses [36,37]. No significant differences in kidney function, as assessed by eGFR between groups were found (Table 2). BMI was lower in Gambian subjects compared to British but not in Chinese subjects. However, proportional LBM and sweat losses may be relatively high in Gambian subjects and expected to increase rather than decrease the *u*Cr concentration [38]. Many statistical comparisons were reported in this study. Although Scheffé post-hoc tests were used to minimise the effects of multiple testing, case findings may have occurred. The main finding of this study, i.e. a difference in renal excretion between groups, was however highly significant (*P* < 0.001).

In conclusion, we have demonstrated differences in PTH secretion and in bone and mineral metabolism in response to oral P loading between British, Chinese and Gambian subjects, but no evidence in support of resistance to the resorbing effects of PTH in any group. Further investigations are in progress to explore the underlying mechanisms for the different renal and skeletal response when Ca-P homeostasis is perturbed and the differences in renal P clearance as influenced by FGF23 and kidney function.

## Figures and Tables

**Table 1:** Characteristics of subjects.

	Males			Females		
	British	Gambian	Chinese	British	Gambian	Chinese
Age (years)	67.4 ± 3.3	66.5 ± 3.3	64.4 ± 4.3	65.7 ± 3.9	67.5 ± 4.1	62.5 ± 3.3^gf^⁎
Height (cm)	174.3 ± 5.4	166.0 ± 8.6^bm^⁎⁎	167.2 ± 5.5^bm^⁎⁎	160.7 ± 6.3	157.9 ± 6.4	154.4 ± 4.7^bf^⁎
Weight (kg)	80.7 ± 12.4	58.5 ± 7.8^bm^⁎⁎	71.2 ± 9.1^bm^⁎^,^^gm^⁎⁎	71.6 ± 13.0	55.9 ± 9.8^bf^⁎⁎	62.1 ± 8.0^bf^⁎
BMI (kg/m^2^)	26.5 ± 3.81	21.2 ± 1.57^bm^⁎⁎	25.5 ± 3.17^gm^⁎	27.6 ± 4.02	22.3 ± 1.57^bf^⁎⁎	26.0 ± 2.53
Ca intake (mg/day)	1185 ± 196	367 ± 129^bm^⁎⁎	588 ± 196^bm^⁎⁎^,^^gm^⁎⁎	1151 ± 242	296 ± 140^bf^⁎⁎	509 ± 266^bf^⁎⁎^,^^gf^⁎⁎
P intake (mg/day)	1692 ± 271	803 ± 264^bm^⁎⁎	1303 ± 263^bm^⁎⁎^,^^gm^⁎⁎	1517 ± 224	665 ± 269^bf^⁎⁎	1029 ± 262^cm^⁎^,^^bf^⁎⁎^,^^gf^⁎⁎
Ca:P	0.70 ± 0.08	0.46 ± 0.08^bm^⁎⁎	0.45 ± 0.11^bm^⁎⁎	0.76 ± 0.13	0.45 ± 0.12^bf^⁎⁎	0.48 ± 0.15^bf^⁎⁎
Femoral neck BMD (g/cm^2^)	0.947 ± 0.121	0.916 ± 0.138	0.898 ± 0.114	0.833 ± 0.069	0.749 ± 0.094	0.774 ± 0.118
Femoral trochanter BMD (g/cm^2^)	0.889 ± 0.113	0.804 ± 0.117	0.824 ± 0.116	0.685 ± 0.082	0.647 ± 0.078	0.671 ± 0.084

Means (± SD). Differences were examined by Scheffé post-hoc tests. Country differences were examined within gender group and sex differences examined within country. ^bm, bf, cm, cf, gm, gf^ next to a value indicates significant difference from counterpart(s) in other column(s). ⁎*P* < 0.05; ⁎⁎*P* < 0.01. Ca and P intakes were assessed on day 0 for each season and were presented as means of two seasons. BMI, body mass index; Ca, calcium; P, phosphorus, BMD, bone mineral density.

**Table 2:** Baseline plasma and urinary concentrations of calcium and phosphate, calciotropic hormones and markers of bone metabolism and kidney function indicators.

	Males			Females		
	British	Gambian	Chinese	British	Gambian	Chinese
*i*Ca (mg/dl)	4.72 ± 0.12	4.68 ± 0.12	4.68 ± 0.12	5.04 ± 0.16	4.88 ± 0.2^gm^⁎	4.72 ± 0.12
Ca (mg/dl)^a^	9.28 ± 0.36	8.60 ± 0.56	9.52 ± 0.44^gm^⁎⁎	9.28 ± 0.56	9.16 ± 0.60	9.64 ± 0.44
P (mg/dl)	2.72 ± 0.37	3.25 ± 0.37^bm^⁎⁎	3.19 ± 0.40^bm^⁎	3.40 ± 0.28^bm^⁎⁎	3.52 ± 0.46	3.59 ± 0.49
*u*Ca/*u*Cr	0.29 ± 0.14	0.23 ± 0.19	0.31 ± 0.14	0.46 ± 0.25	0.26 ± 0.29^bf^⁎	0.42 ± 0.19^gf^⁎
*u*P/*u*Cr	1.73 ± 0.47	1.41 ± 0.44	1.65 ± 0.44	2.16 ± 0.70	1.82 ± 0.50	1.97 ± 0.67
25-OHD (ng/ml)	21.9 ± 7.2	25.7 ± 6.2	17.9 ± 8.9^gm^⁎⁎	25.4 ± 5.8	29.1 ± 7.0	18.6 ± 6.5^bf^⁎^,^^gf^⁎⁎
1,25(OH)_2_D (pg/ml)	40.1 ± 7.8	60.3 ± 13.1^bm^⁎⁎	40.0 ± 10.2^gm^⁎⁎	39.1 ± 9.5	83.8 ± 22.4^gm^⁎⁎^,^^bf^⁎⁎	45.5 ± 9.7^gf^⁎⁎
PTH (ng/l)	31.2 (27.8, 34.9)	45.5 (41.3, 50.0)^bm^⁎	31.5 (29.3, 33.9)^gm^⁎	33.8 (29.7, 38.4)	51.8 (46.5, 57.7)^bf^⁎⁎	36.6 (32.3, 41.4)^gf^⁎
PINP (ng/ml)	34.0 (29.2, 39.5)	54.8 (48.9, 61.5)	36.0 (30.2, 42.9)	48.4 (43.0, 54.8)	83.6 (68.8, 101.5)^bf^⁎	41.8 (37.2, 47.0)^gf^⁎⁎
OC (ng/ml)	19.4 (17.4, 21.5)	30.6 (27.8, 33.7)	17.1 (14.5, 20.4)^gm^⁎⁎	29.3 (26.4, 32.5)	44.7 (36.9, 54.3)	20.9 (18.7, 23.3)^gf^⁎
BALP (U/l)	22.3 (20.4, 24.3)	26.6 (24.4, 29.0)	23.1 (19.9, 26.9)	26.8 (24.9, 28.8)	37.5 (31.4, 44.9)	25.0 (22.8, 27.4)^gf^⁎
CTX_β_ (ng/ml)	0.26 (0.22, 0.32)	0.64 (0.56, 0.73)^bm^⁎⁎	0.31 (0.25, 0.39)^gm^⁎⁎	0.44 (0.35, 0.47)	0.73 (0.62, 0.86)	0.42 (0.37, 0.48)
*u*DPD/*u*Cr	5.25 (4.75, 5.79)	8.08 (7.24, 9.06)	3.53 (2.93, 4.28)^bm^⁎^,^^gm^⁎⁎	8.52 (7.69, 9.42)^bm^⁎	12.43 (10.95, 14.35)^gm^⁎	6.36 (5.81, 6.98)^cm^⁎⁎^,^^gf^⁎⁎
eGFR (ml/min)	76 ± 18.1	66 ± 15.1	79 ± 18.6	66 ± 25.4	67 ± 21.6	71 ± 16.6
TmP/GFR (mg/dl)	2.49 ± 0.55	3.12 ± 0.52^bm^⁎	2.98 ± 0.60	2.84 ± 0.39	3.33 ± 0.53^gm^⁎	3.90 ± 0.77^bf^⁎⁎^,^^cm^⁎⁎

Values are mean ± SD or geometric means (95% confidence intervals). Differences were examined by Scheffé post-hoc tests. Country differences were examined within gender group and sex differences examined within country. ^bm, bf, cm, cf, gm, gf^ next to a value indicates significant difference from counterpart(s) in other column(s). ⁎*P* < 0.05; ⁎⁎*P* < 0.01. *u*, urinary concentration; Ca, calcium; P, phosphorus; *i*Ca, ionized calcium; Cr, creatinine; 25-OHD, 25 hydroxyvitamin D; 1,25(OH)_2_D, 1,25 dihydroxyvitamin D; P1NP, total N-terminal propeptides of type 1 procollagen; OC, osteocalcin; BALP, bone alkaline phosphatase; βCTX, β form of cross-linked C-telopeptide of type 1 collagen; DPD, deoxypyridinoline; eGFR, estimated glomerular filtration rate; TmP/GFR the ratio of the maximum tubular reabsorption rate of phosphate to glomerular filtration rate. All biochemical markers are presented as means of timepoint A on days 0 and 1 and are averaged over two seasons with the exception of *p*25-OHD that was only measured and eGFR and TmP/GFR that were calculated on the basis of corresponding values in blood and urine, respectively at timepoint A on day 0 in each season.

a The comparison for *p*Ca was adjusted for *p*Albumin by including it as one of the independent variables in the analysis.

## References

[1] Riggs B.L., Melton L.J. (1986). III Involutional osteoporosis. N. Engl. J. Med..

[2] Adebajo A.O., Cooper C., Evans J.G. (1991). Fractures of the hip and distal forearm in West Africa and the United Kingdom. Age Ageing.

[3] Aspray T.J., Yan L., Prentice A. (2005). Parathyroid hormone and rates of bone formation are raised in perimenopausal rural Gambian women. Bone.

[4] Yan L., Zhou B., Prentice A., Wang X., Golden M.H. (1999). Epidemiological study of hip fracture in Shenyang, People's Republic of China. Bone.

[5] Yan L., Zhou B., Wang X., D'Ath S., Laidlaw A., Laskey M.A. (2003). Older people in China and the United Kingdom differ in the relationships among parathyroid hormone, vitamin D, and bone mineral status. Bone.

[6] Bates C.J., Carter G.D., Mishra G.D., O'Shea D., Jones J., Prentice A. (2003). In a population study, can parathyroid hormone aid the definition of adequate vitamin D status? A study of people aged 65 years and over from the British National Diet and Nutrition Survey. Osteoporos. Int..

[7] Jarjou L.M., Prentice A., Sawo Y., Laskey M.A., Bennett J., Goldberg G.R. (2006). Randomized, placebo-controlled, calcium supplementation study in pregnant Gambian women: effects on breast-milk calcium concentrations and infant birth weight, growth, and bone mineral accretion in the first year of life. Am. J. Clin. Nutr..

[8] Khaw K.T., Sneyd M.J., Compston J. (1992). Bone density parathyroid hormone and 25-hydroxyvitamin D concentrations in middle aged women. BMJ.

[9] Cosman F., Morgan D.C., Nieves J.W., Shen V., Luckey M.M., Dempster D.W. (1997). Resistance to bone resorbing effects of PTH in black women. J. Bone. Miner. Res..

[10] Fuleihan G.E., Gundberg C.M., Gleason R., Brown E.M., Stromski M.E., Grant F.D. (1994). Racial differences in parathyroid hormone dynamics. J. Clin. Endocrinol. Metab..

[11] Cosman F., Shen V., Morgan D., Gordon S., Parisien M., Nieves J. (2000). Biochemical responses of bone metabolism to 1,25-dihydroxyvitamin D administration in black and white women. Osteoporos. Int..

[12] Silverberg S.J., Fitzpatrick L.A., Bilezikian J.P., Marcus R, Feldman D, Kelsey J (1996). The role of parathyroid hormone and vitamin D in the pathogenesis of osteoporosis. Osteoporosis.

[13] Silverberg S.J., Shane E., de la Cruz L., Segre G.V., Clemens T.L., Bilezikian J.P. (1989). Abnormalities in parathyroid hormone secretion and 1,25-dihydroxyvitamin D3 formation in women with osteoporosis. N. Engl. J. Med..

[14] Bodner C.H., Soutar A., New S.A. (1998). Validation of a food frequency questionnaire for use in a Scottish population: correlation of antioxidant vitamin intakes with biochemical measures. J. Hum. Nutr. Dietet..

[15] Yan L., Zhou B., Greenberg D., Wang L., Nigdikar S., Prynne C. (2004). Vitamin K status of older individuals in northern China is superior to that of older individuals in the UK. Br. J. Nutr..

[16] Cole T.J. (2000). Sympercents: symmetric percentage differences on the 100 log(e) scale simplify the presentation of log transformed data. Stat. Med..

[17] Barth J.H., Jones R.G., Payne R.B. (2000). Calculation of renal tubular reabsorption of phosphate: the algorithm performs better than the nomogram. Ann. Clin. Biochem..

[18] Payne R.B. (1998). Renal tubular reabsorption of phosphate (TmP/GFR): indications and interpretation. Ann. Clin. Biochem..

[19] Heaney R.P. (2004). Phosphorus nutrition and the treatment of osteoporosis. Mayo. Clin. Proc..

[20] Bijvoet O.L.M., Massry S, Fleisch H (1980). Indices for the measurement of the renal handling of phosphate. Renal handling of phosphate.

[21] Mirams M., Robinson B.G., Mason R.S., Nelson A.E. (2004). Bone as a source of FGF23: regulation by phosphate?. Bone.

[22] Berndt T., Kumar R. (2007). Phosphatonins and the regulation of phosphate homeostasis. Annu. Rev. Physiol..

[23] Nishida Y., Taketani Y., Yamanaka-Okumura H., Imamura F., Taniguchi A., Sato T. (2006). Acute effect of oral phosphate loading on serum fibroblast growth factor 23 levels in healthy men. Kidney. Int..

[24] Martin D.R., Ritter C.S., Slatopolsky E., Brown A.J. (2005). Acute regulation of parathyroid hormone by dietary phosphate. Am. J. Physiol. Endocrinol. Metab..

[25] DeLuca H.F. (2004). Overview of general physiologic features and functions of vitamin D. Am. J. Clin. Nutr..

[26] Charles P., Eriksen E.F., Hasling C., Sondergard K., Mosekilde L. (1991). Dermal, intestinal and renal obligatory losses of calcium: relation to skeletal calcium loss. Am. J. Clin. Nutr..

[27] Karkkainen M., Lamberg-Allardt C. (1996). An acute intake of phosphate increases parathyroid hormone secretion and inhibits bone formation in young women. J. Bone Miner. Res..

[28] Kemi V.E., Karkkainen M.U., Lamberg-Allardt C.J. (2006). High phosphorus intakes acutely and negatively affect Ca and bone metabolism in a dose-dependent manner in healthy young females. Br. J. Nutr..

[29] Jilka R.L. (2007). Molecular and cellular mechanisms of the anabolic effect of intermittent PTH. Bone.

[30] Lian J.B., Stein G.S., Aubin J.E., Favus MJ (2003). Bone formation: maturation and functional activity of osteoblast lineage cells. Primer on the metabolic bone diseases and disorders of mineral metabolism.

[31] Kurahara N., Hosoda K., Tatsumi J. (1998). The N-terminal fragment of osteocalcin is released during osteoclastic bone resorption in vitro. J. Bone Miner. Res..

[32] de Francisco A.M., Riancho J.A., Amado J.A., del Arco C., Macias J.G., Cotorruelo J.G. (1987). Calcium, hyperparathyroidism, and vitamin D metabolism after kidney transplantation. Transplant. Proc..

[33] Portale A.A., Halloran B.P., Murphy M.M., Morris R.C. (1986). Oral intake of phosphorus can determine the serum concentration of 1,25-dihydroxyvitamin D by determining its production rate in humans. J. Clin. Invest..

[34] Kemp G.J., Blumsohn A., Morris B.W. (1992). Circadian changes in plasma phosphate concentration, urinary phosphate excretion, and cellular phosphate shifts. Clin. Chem..

[35] Schlemmer A., Hassager C. (1999). Acute fasting diminishes the circadian rhythm of biochemical markers of bone resorption. Eur. J. Endocrinol..

[36] Welle S., Thornton C., Totterman S., Forbes G. (1996). Utility of creatinine excretion in body-composition studies of healthy men and women older than 60 y. Am. J. Clin. Nutr..

[37] Walser M. (1987). Creatinine excretion as a measure of protein nutrition in adults of varying age. J. Parenter. Enteral. Nutr..

[38] Singh J., Prentice A.M., Diaz E., Coward W.A., Ashford J., Sawyer M. (1989). Energy expenditure of Gambian women during peak agricultural activity measured by the doubly-labelled water method. Br. J. Nutr..

[39] Traynor J., Mactier R., Geddes C.C., Fox J.G. (2006). How to measure renal function in clinical practice. BMJ.

